# Path Planning of Mobile Robot With Improved Ant Colony Algorithm and MDP to Produce Smooth Trajectory in Grid-Based Environment

**DOI:** 10.3389/fnbot.2020.00044

**Published:** 2020-07-09

**Authors:** Hub Ali, Dawei Gong, Meng Wang, Xiaolin Dai

**Affiliations:** School of Mechanical and Electrical Engineering, University of Electronic Science and Technology of China, Chengdu, China

**Keywords:** mobile robot, ant colony algorithm, Markov decision process model, motion planning, obstacle avoidance

## Abstract

This approach has been derived mainly to improve quality and efficiency of global path planning for a mobile robot with unknown static obstacle avoidance features in grid-based environment. The quality of the global path in terms of smoothness, path consistency and safety can affect the autonomous behavior of a robot. In this paper, the efficiency of Ant Colony Optimization (ACO) algorithm has improved with additional assistance of A* Multi-Directional algorithm. In the first part, A* Multi-directional algorithm starts to search in map and stores the best nodes area between start and destination with optimal heuristic value and that area of nodes has been chosen for path search by ACO to avoid blind search at initial iterations. The path obtained in grid-based environment consist of points in Cartesian coordinates connected through line segments with sharp bends. Therefore, Markov Decision Process (MDP) trajectory evaluation model is introduced with a novel reward policy to filter and reduce the sharpness in global path generated in grid environment. With arc-length parameterization, a curvilinear smooth route has been generated among filtered waypoints and produces consistency and smoothness in the global path. To achieve a comfort drive and safety for robot, lateral and longitudinal control has been utilized to form a set of optimal trajectories along the reference route, as well as, minimizing total cost. The total cost includes curvature, lateral and longitudinal coordinates constraints. Additionally, for collision detection, at every step the set of optimal local trajectories have been checked for any unexpected obstacle. The results have been verified through simulations in MATLAB compared with previous global path planning algorithms to differentiate the efficiency and quality of derived approach in different constraint environments.

## Introduction

For decades, the concept of autonomous driving has become familiar with the public due to its vast applications in multi-dimensional aspects (Gu et al., 2017). Therefore, ambitious research has taken place to improve the autonomous behavior of mobile robots. Mobile robots have applications in different domains such as industry, military, transportation, etc. (Duchoe et al., 2014). In industries, autonomous mobile robots are used in logistics warehousing, flexible manufacturing, and intelligent inspection are much more efficient than humans and are available at a relatively lower cost. In mobile robot autonomous behavior is achieved through perception, decision, and actions (Sarkar et al., 2018). Perception contains sensor data from the environment, which has been actuated to low-level control according to decision ability. The decision stage is known as the motion-planning stage. Many approaches have been introduced to improve the motion-planning ability of mobile robots. Motion planning is a middle stage between perception and actuation and is responsible for the autonomous behavior of a robot. Motion planning is mainly categorized into Global Path Planning (GPP) and Local Path Planning (LPP) according to problem estimation.

GPP is a key technique for robots to acquire an optimal route from the initial position to the final destination. Several different approaches have introduced to acquire an optimal global path. Most GPP techniques use discrete search optimization and has applied in the grid-based environment. The path obtained in the grid map consists of suboptimal points connected though straight line segments and, the path contains sharp bends. The bends present in the global path can cause a jerky behavior in a robot's motion and, instant change in velocity and acceleration also effect on energy consumption of robots. In contrast, a smooth route can deliver a mobile robot a comfortable and safe drive. Therefore, the curvilinear global route has considered a suitable choice for better navigation and used in real-time scenarios. For example, In Defense Advanced Research Projects Agency (DARPA) autonomous challenge (2012), a vehicle used the pre-defined curvilinear route (Chu et al., 2012). The global path planning techniques including simulated annealing algorithm (Miao and Tian, 2013), potential function theory (Cetin and Yilmaz, 2014) has considered as traditional approaches due to their limited functionalities. The genetic algorithm has applied to get the global path in Bakdi et al. (2017), but it shows poor computation ability in complex maps. On the other hand, Ant Colony Optimization (ACO) (Wang et al., 2016), genetic algorithm (Huang and Fei, 2018), neural network (He et al., 2015, 2017), and particle swarm algorithm (Song et al., 2017) have considered as intelligent approaches. ACO has considered as one of the popular evolutionary approaches to solve optimization problems. Due to its advantages, such as good feedback information, robustness, better-distributed computing (Akka and Khaber, 2018) and, the ability to be easily combined with many path-planning approaches, it is often used to deliver an optimal solution of global path planning issues.

Similarly, LPP has applied to localize the robot's motion along a global path to avoid unknown obstacles. The predefined global waypoints have given to the robot in various LPP techniques and, it has to maintain an offset position on the centerline to avoid dynamic obstacles. Meanwhile, it plans smooth trajectories to move forward toward destination. DAPRA international auto-driving challenge has organized to present many state of the art technologies to achieve autonomous smooth driving features. Sampling-based approach as RRT^*^ has been used in path planning (Hwan Jeon et al., 2013). the algorithm used in Pivtoraiko and Kelly (2005) is a discrete representation of the planning area with a grid of states (usually a hyper-dimensional one). Reinforcement learning has been applied in a hierarchical path planning approach to achieve local planning and navigation (Zuo et al., 2015). Different approaches have advantages as well as drawbacks in different complex situations. Such as few mentioned techniques provide local path planning features and, few can only work in global path planning.

## Contribution

The motivation comes from the research work presented in state-of-art literature to introduce a combined approach for enhancing the path planning abilities of a mobile robot in a known and unknown statics constraint environment. The global trajectory obtained through pre-defined waypoints in local path planning algorithms like (Walambe et al., 2016; Hu et al., 2018). This approach delivers global and local trajectory planning features. For global path planning an improved ACO version has presented that deliver an optimal trajectory with efficient computational ability. The ACO has enhanced with A* multi-directional algorithm. In ACO, starting iterations don't have pheromone concentration and ants have to move randomly (blind search) to reach a goal position. This makes it computationally expensive and time-consuming. Hence, the ant colony algorithm needs to improve its computational efficiency. Therefore, A* Multi-directional algorithm is introduced to sort the area of nodes having a high possibility of obtaining an optimal global path. ACO utilized that information of A* multi-directional algorithm as closest area for search and allow ants to move in that directions. It increases the efficiency of ACO to deliver global path in complex maps efficiently. The quality of path has improved using MPD trajectory evaluation model. ACO gives a sequence of optimal grids and each grid is represented by its center point and the global path consists of straight-line segments containing sharp bends formed through connecting grid points in sequential order. Trajectory evaluation model filter the path points and arc-length parametrization bring path consistency and smoothness in final trajectory. To attain optimal driving behavior, a set of lateral and longitudinal coordinates has been generated. Lateral coordinates refer to offset distance d from reference points to navigate, whereas longitudinal coordinates represent distance covered at each waypoint in sense of cumulative arc length s, respectively. Both lateral and longitudinal coordinates are unified to produce a feasible set of trajectories along the reference path. Each forward move of the robot on the reference path will generate a set of trajectories to avoid unexpected obstacles. Every trajectory has to checked with defined cost constraints that include curvature limit, speed limit, and obstacle check.

## Related Work

In recent years, tremendous research efforts have made for improving the path planning efficiency of mobile robots in both global and local path planning techniques. For global path planning, ACO has chosen in this paper because of its advantages. Although, ACO has drawbacks of slow convergence and pheromone update. To address this problem, many approaches have been proposed (Stützle and Hoos, 2000; Zeng et al., 2016; Zhao et al., 2016). Pheromone rate has been updated after each successful iteration of ant in ACO to improve the convergence rate (Zhao et al., 2016). In Zeng et al. (2016) convergence rate with search ability has been increased through upgrading pheromone update formula and adaptively varying volatilization rate. To avoid blind search in Stützle and Hoos (2000) an initial path has produced and transformed into initial pheromone distribution in ACO. Geometric method has been introduced to optimize the global route and also local diffusion of pheromones has obtained from a force factor defined in artificial potential field to enhance the ability of obstacle detection (Liu et al., 2017). In Yen and Cheng (2018), fuzzy logic is combined with ACO to reduce repetitive learning errors. In Long et al. (2019), A* Heuristic characteristics improved ACO optimization performance in various complexity maps. In the discrete-search algorithm, linear interpolation has been performed to bring smoothness in the global path (Ferguson and Stentz, 2006). In Zuo et al. (2015), the MDP model has been used with the A* algorithm to achieve a smooth path and improve navigation. These approaches can improve the efficiency of ACO. However, the quality of the path obtained in the grid environment doesn't match with the dynamic properties of a mobile robot due to its roughness and sharp bends. In the local path search, a curvilinear road is formed with cubic spline interpolation and a set of feasible trajectories has been generated along the roadside to avoid static obstacles (Chu et al., 2012). In Hu et al. (2018), curvilinear road shape has been produced from predefined waypoints and model predictive approach is used to avoid static and moving obstacles. Similarly, lateral and longitudinal movements have been introduced within the steer relative coordinates to achieve optimal motion control (Werling et al., 2012). The global reference path is derived from the vision map using the lane-level accurate localization information via the LiDAR-based localization methods (Hwang et al., 2003; Li et al., 2017). In Li et al. (2015), conjugate gradient non-linear optimization and cubic spline curve are used to achieve a smooth global path from digital map and curvilinear coordinates framework is used to obtain optimal trajectories. Likewise, these techniques can efficiently handle local path problem but do not work well to achieve optimal global path in complex constraints environment.

## Reference Path Generation in Static Constraints Environment

Global Path Planning is used to obtain a global route from an initial position of the robot to destinations in a static constraint environment. GPP is an essential technique for a mobile robot to find a suitable path in various situations. It is simulated in a grid-based environment with prior knowledge of static constraints. In this section, the grid model has presented and the serial number method has used to reduce complexity. Multi-Directional A* algorithm has utilized to get the initial searching area, that assist the ACO to get final optimal path. A constraints policy is presented for initial search of ants in ACO and evaluation function of A* algorithm is utilized to accelerate convergence speed in search strategy of ant colony algorithm with better heuristic information. Global search ability has enhanced by MAX_MIN ant system through updating path pheromone information. Moreover, the Trajectory evaluation model has introduced to increase the visibility of the ACO path and filter the corner points through MDP.

### Environment Model

**Figure 1:**
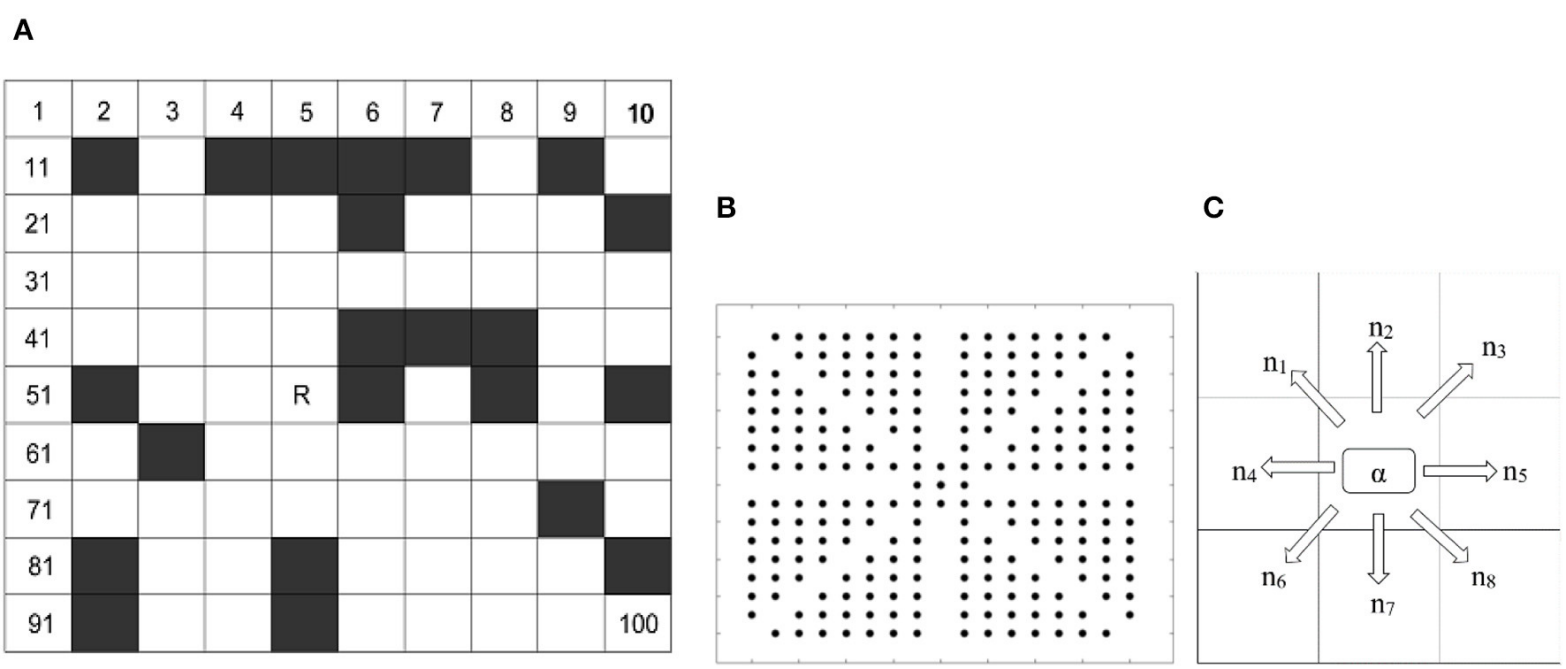
**(A)** Grid model **(B)** Possible visiting nodes around center node **(C)** Possible visiting direction for ant.

This path planning approach is implemented in a grid-based environment. The grid method is simple and effective to create and maintain the grid model. Moreover, the grid method has strong adaptability for obstacles and also convenience for computer storage and processing. It divides the working space into *N* × *N* squares. As shown in Figure 1A, white grids are spaces where robot can move freely, in contrast black grids represent constraints area. In order to identify constraint areas, the white grid cell is represented with 0, and black grid unit is represented with 1.

The grid model is consisting of a two-dimensional coordinate system. Each grid is marked according to the serial number method. In *N* × *N* grid map, the starting node is named after Start and the target node is named after Destination. The position coordinates (*x, y*) corresponding to any grid whose grid number is *R* is as follow:

(1) $$\{\begin{array}{l} x=\;\{\begin{array}{ll} mod\left(R,N\right)-0.5 & if\;mod\left(R,N\right)!=0\\ \;N+mod\left(R,N\right)-0.5 & \;otherwise \end{array}\\ y=N+0.5-ceil(\frac{R}{N}) \end{array}$$

The serial number method is applied to reduce the computational complexity of ACO. The direction of each move from the grid's center point to neighbor grids has been simplified with arithmetic operation. In Figure 1C, α represents the number of a central grid point and *N* is the number of rows and columns of the grid map. *n* represents all possible the direction.

### A* Multi-Directional Algorithm (A*MDA)

**Figure 2:**
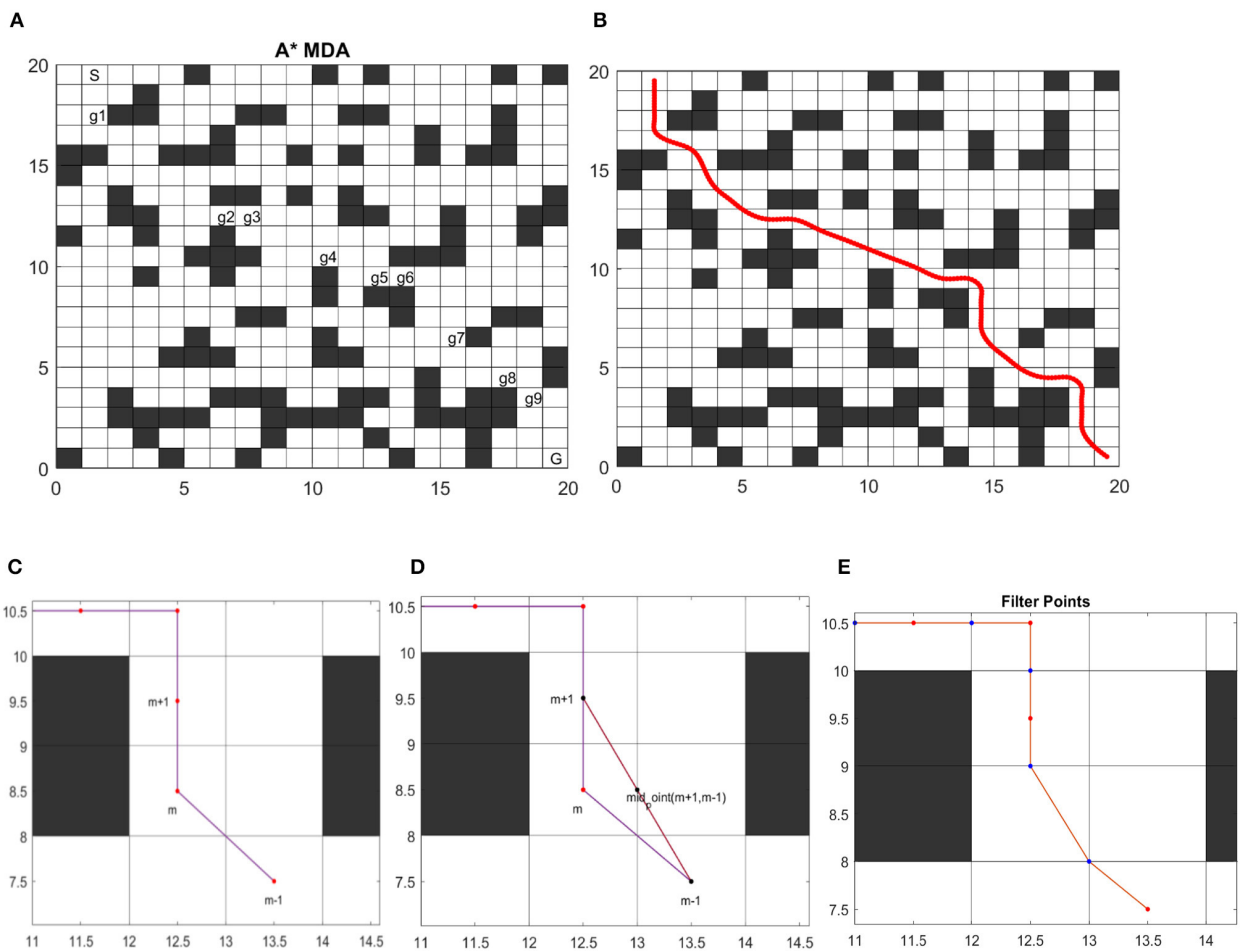
Shows the steps for global path optimizing. **(A)** A* MDA search for ACO. **(B)** Final trajectory with arc-length parameterization. **(C)** MDP model has been applied between two points. **(D)** Evaluation of mid-point according to cost policy. **(E)** The bad point has removed from the path after evaluation.

In ACO, ants start to search in the map and after every successful iteration, a new pheromone has updated in the network that represents a specific direction toward a goal. After a few iterations, the ants start to converge their directions according to a higher pheromone ratio. At starting iterations, the ants do not have proper guidance through the pheromone ratio. Therefore, they move in different directions to seek a goal node and if the map has a large and complex search space then it consumes more time. A* algorithm has been introduced with multi-direction path search features to assist the ACO. Figure 1B has shown possible connecting nodes with the center node. Each node has to be estimated with total cost value (*n*), which is the sum of *g*(*n*) and *h*(*n*). It defines two matrices to list and mark visited node named as Open_ list and Closed_list. Open_list is marked with visited nodes and Closed_list contains the record of obstacles and previously selected nodes to avoid repetition. The next node selection is based on the lowest cost value of *f*(*n*). It continues to search, until the destination node arrives. To retrieve the best nodes, the location of each parent node is stored into ParentX and ParentY. This algorithm provides multi-goals on short distances between start and actual destination. Meanwhile, the best nodes selected by A* Multi-Directional algorithm are not enough to complete a global path individually. Therefore, ACO has been utilized to fulfill the missing nodes and complete a global path. Figure 2A shows that the multi-goals generated by A*MDA and Figure 2B shows the final trajectory. Table 1 provides algorithm flow:

(2) $$\begin{array}{r} f\left(n\right)=g\left(n\right)+h\left(n\right) \end{array}$$

**Table 1:** A* Multi-directional algorithm.

**Procedure:** A*MDA
Initialization: Start(S), Goal(E), MAP, Open_list, Closed_list, PerentX_list, PerentY_list
// Setting up matrices Q representing neighbors to be investigated
// Add the start node
put the start Node on the Open_list (it's *f*(*S*) equals to *h*(*s*))
// Loop until you find the goal node
**While** the current node is equal to goal node
//Initializing current node with min *f*(*n*) value and opening first node
**if** *f*(*n*) equals to infinity
// Open_list is empty or goal not found
**Break; //** path not found
**end if**
**for** Current node equals to goal node
**Break;** // Retrieve the path
**end for**
// visit the Q neighbors and calculate *f*(*n*) for each
**for** Q **do**
Using Equation (2) calculate *f*(*n*)
// save visited nodes x, y values in PerentX_list and PerentY_list.
**end for**
**end While**
**While //** Retrieve the path,
Currentnode_X = ParentX(Current node);
Currentnode_Y = PerentY(Current node);
**If** current node is equal to goal node
// Output = {Start, goal*n*, goal*n*_+_1, goal*n*_+_2,…goal_*t*_}
**end if**
**end while**

### Improved ACO With Initial Constraints Policy

ACO has been applied to achieve optimal global path from an initial position to destination in a grid-based environment. A* Multi-directional algorithm provides the direction guideline to ACO. The ants in traditional ACO have to search all possible grids and every next grid is decided by the roulette wheel method and repeated until the target point is obtained. It was computationally expensive. According to the grid-based environment obstacle grid represented with 1 and free grid represent with 0. In absence of any constraints, Ant α can move in 8 directions from the center grid as shown in Figure 1C. In the situation of obstacle detection, the remaining direction grids can be chosen using heuristic information. Initial constraints policy defines a cost for eliminating a specific direction grid, which does not maintain offset distance with obstacle grids. Improved A* heuristic characteristics are used to improve efficiency. To improve premature convergence and phenomenon update strategy MAX_MIN, the ant system has been utilized. The improvements are given as below:

#### Initial Constraint Policy

Initial constraints policy is used in case of obstacle detection. When ant α found an obstacle, it has to turn toward remaining directions. Initial constraint policy limits the ant to consider those grids which do not maintain offset distance with obstacle grid. With regards to serial number system, an ant α has 8 neighbor grids directions presented in Table 2. Constraints policy is formed based on obstacle grid location and neighbor grids. In Table 3, on every obstacle direction constraint policy eliminates following grids to maintain offset distance in the final global path.

**Table 2:** Arithmetic equations for direction.

**Direction**	**Arithmetic operation**
n1	α – (N+1)
n_2_	α – N
n3	α – (N−1)
n4	α – 1
n5	α + 1
n6	α + (N−1)
n7	α + N
n8	α + (N + 1)

**Table 3:** Initial constraints policy for ant.

**Obstacle direction**	**Eliminating grids**
n_2_	n1 & n3
n4	n1 & n6
n7	n6 & n8
n5	n3 & n8

#### Heuristic Information and Path Strategy Information

In ACO, poor pheromone distribution at initial steps of ant search causes slow convergence and increase search time. The direction information and path strategy of ACO has been improved with A* algorithm characteristics to avoid blind search at initial steps of ant (Duchoe et al., 2014). The estimated function of A* has been used to enhance directional information expressed as Equation (2). *g*(*n*) represents distance from source grid to current grid and *h*(*n*) represents distance from current grid to destination grid, respectively.

In order to achieve reduced number of sharp bending in global path, a bending reducing operator has been derived in Equation (3) and included in heuristic search information of ACO. *cost*(*bend*) is bending reducing operator. Number of moves from previous node to next node represented with *turn* and *theta* contains the angle formed among previous node and next node with respect to current node. φ and ψ represents cost of transforming turning times and angle into grid length, respectively.

(3) $$\begin{array}{l} cost\left(bend\right)=\varphi^{*}turn+\psi^{*}thita\\ \;\eta_{ij}\left(t\right)=\frac{Q_{2}}{f\left(n\right)+cost\left(bend\right)} \end{array}$$

#### MAX_MIN Ant System (MMAS)

MAX_MIN system has been used in Stützle and Hoos (2000) to enhance pheromone strategy in ACO. Pheromone is updated after each iteration in conventional ACO, with MMAS the optimum route pheromone has been updated to the pheromone trial exclusively presented in Equation (4). *Q*_1_, *Q*_2_ are coefficient and each contains constant value less than one. *L*^*best*^contains cost value for present shortest route. *Cals*(*l*) and *Turns*(*l*) represents sum of bending angles changed in path and sum of turns for optimal route. Similarly, ω_1_ and ω_2_ are considered as weights and defined according to robot's structure (Wu et al., 2011).

(4) $$\begin{array}{l} \tau_{ij}\left(t+1\right)=\left(1-\rho \right)\tau_{ij}\left(t\right)+\Delta \tau_{ij}^{best}\\ \;\Delta \tau_{ij}^{k}\left(t\right)=\frac{Q_{1}}{L^{best}}+\frac{Q_{3}}{\omega_{1}Cals\left(l\right)+\omega_{2}Turns\left(l\right)} \end{array}$$

In conventional ACO, an ant during search may fall into local optima and mislead the search process. In this context, MMAS pheromone trials has been bounded to upper limits and lower limits [τ_min_,τ_max_,] as:

(5) $$\left\{ \begin{array}{l} \tau_{\min},\tau \leq \tau_{\min}\\ \;\tau ,\tau_{\min}<\tau \leq \tau_{\max}\\ \tau_{\max},\tau >\tau_{\max} \end{array}\right.$$

The above procedure aims to optimize the performance of ACO.

### Markov Decision Process (MDP) Model With Novel Reward Policy

MDP consists of state-action transition model, which takes sequence of optimal path grids and evaluate every grid with respect to defined reward policy. Each optimal path grid is referred to as state and possible action has been taken based on reward policy. Reward policy is designed in order to check every middle point and its direction with respect to first two neighbor grids. Global route achieved through the Improved Ant colony algorithm. ACO is applied in a grid-based environment to achieve a sequence of optimal grids points in the (x, y) coordinates. These center points are known as path candidates. The global route produced by path candidates consists of straight suboptimal line segments that contain sharp bending and the result is a rough path shown in Figure 2C. To deal with the non-holonomic properties of robot, this path is not feasible to maintain a smooth and safe drive. To achieve path consistency and smoothness a novel evaluation function is introduced. This evaluation function examines path point sequence and remove bad path candidates according to the given cost policy. The evaluation technique follows the given steps:

#### Linear Interpolation

To increase the visibility of global path extra points has generated through Linear interpolation. Linear interpolation has a broad area of applications (Zheng et al., 2012). In Chapra (2012), interpolating function *f*(*x*) is used to generate new point between every two global path points. In Equation (6) (*x*_0_, *y*_0_) and (*x*_1_, *y*_1_) are two consecutive global path points. To compute new points in a straight line, the equation of straight slope is used. In Figure 2C LI is used to generate a midpoint between every two path candidates showed by dot line.

(6) $$\begin{array}{r} f\left(x\right)=y=\frac{y_{1}-y_{2}}{x_{1}-x_{0}}\left(x-x_{0}\right)+y_{0} \end{array}$$

#### Cost Policy

This paper introduces a new cost policy to filter the path obtained in grid environment. Path has considered of points *m*.

$$\begin{array}{l} m_{i}=m_{1},m_{2},m_{3},m_{4},\ldots m_{l}\;and\;l=total\;number\;of\;points \end{array}$$

In order to achieve efficient computation results a novel cost policy is formed to evaluate grid points. The main objective of this evaluation function is to remove path points, which do not satisfy the cost policy. This cost policy is mainly consisting of following steps:

1. The Mid-Point Evaluation method has introduced to analyze the direction of each point among sequence. The evaluation path point has denoted by *m*_*i*_. *m*_*i*_ − 1 and *m*_*i*_ + 1 are the first and second neighbor, respectively. Figure 2D shows the path points obtained using ACO present the sharp corners. Cost policy will perform midpoint evaluation on each grid point to recognize its direction to neighbors.
2. The path points have been passed through MDP evaluation model presented in Table 4. The cost has set 0 and that point will eliminate from path if the midpoint value of first two neighbors is equal to centered point as shown in cost policy Table 5. In Figure 2E the m point doesn't satisfy the cost policy and allotted 0 value, hence deleted from path.

**Table 4:** MDP state-action Model.

**State**	**{m| m ϵ set of optimal path points }**
**Action**	(remove m from path, Keep m in path)
**Reward**	0 is for removed m and 1 is for kept m

**Table 5:** Novel reward policy.

**If direction of first neighbor grids with m**	**Annotation**	**Reward**
Midpoint(*m*_*i*_ − 1&*&m*_*i*_ + 1) ≠ *m*_*i*_ *where i is number of path point*	*m*_*i*_ is a bending point	Assign 0 else 1

#### Arc-Length Parametrization With Cubic Spline

In order to achieve optimal route from initial position to destination, several different approaches have used arc-length parameterization technique in predefined global waypoints to generate a reference road path. In grid-based environment, the obtained global path doesn't contain path consistency and robot is unable to localize itself precisely on derived route. This is considered as a drawback for solving path planning problem in grid-based environment. Therefore, the arc-length parameterization is applied to get curvilinear smooth route from refined path points. Figure 2B shows the smoothness and consistency in path generated with equally spaced points having arc length (s) along global route defined thorough Equation (7) (Wang et al., 2002). The *k*_*r*_ curvature of each point present in optimal reference line has been derived through (8).

(7) $$\begin{array}{l} x_{\text{s}}=a_{\text{x}}{\left(s\right)}^{3}+b_{\text{x}}{\left(s\right)}^{2}+c_{\text{x}}\left(s\right)+d_{\text{x}}\\ y_{s}=a_{y}{\left(s\right)}^{3}+b_{y}{\left(s\right)}^{2}+c_{y}\left(s\right)+d_{y} \end{array}$$

(8) $$k_{r}=\frac{{x_{s}}^{\prime }{y_{s}}^{\prime \prime }-{x_{s}}^{\prime \prime }{y_{s}}^{\prime }}{\sqrt{{\left({x_{s}}^{\prime }+{y_{s}}^{\prime }\right)}^{3}}}$$

*a*_x_, *b*_x_, *c*_x_, *a*_*y*_, *b*_*y*_, *c*_*y*_, *d*_x_, *d*_*y*_ has considered as coefficient. $x_{s}\prime$, $x_{s}\prime \prime$, $y_{s}\prime$, $y_{s}\prime \prime$ are first and second derivatives of *x*_s_, *y*_*s*_ coordinates.

## Unexpected Constraints Avoidance and Trajectory Planning

After obtaining an optimal reference global route, robot starts to move toward destination. Meanwhile, it has to avoid the unexpected constraints and also has to maintain its position on reference trajectory. To obtain optimal behavior in unknown obstacle environments, curvilinear coordinates framework approached human like driving behavior. In human like behavior, distance covered along road and offset to its center is taken into consideration. With this regard, Barfoot and Clark (2004) and Hu et al. (2018) curvilinear road path derived though cubic interpolation between predefined global waypoint. In Li et al. (2015), curvilinear path is adopted from digital map and B-spline curve used to bring smoothness in center line. This paper introduced an efficient approach to derive curvilinear reference route autonomously in complex grid maps using Improved ACO with MDP model and arc length parameterization. To define robot motion on global reference line, lateral and longitudinal coordinates generated and unified to produce set optimal trajectories.

### Formation of Lateral and Longitudinal Coordinates

**Figure 3:**
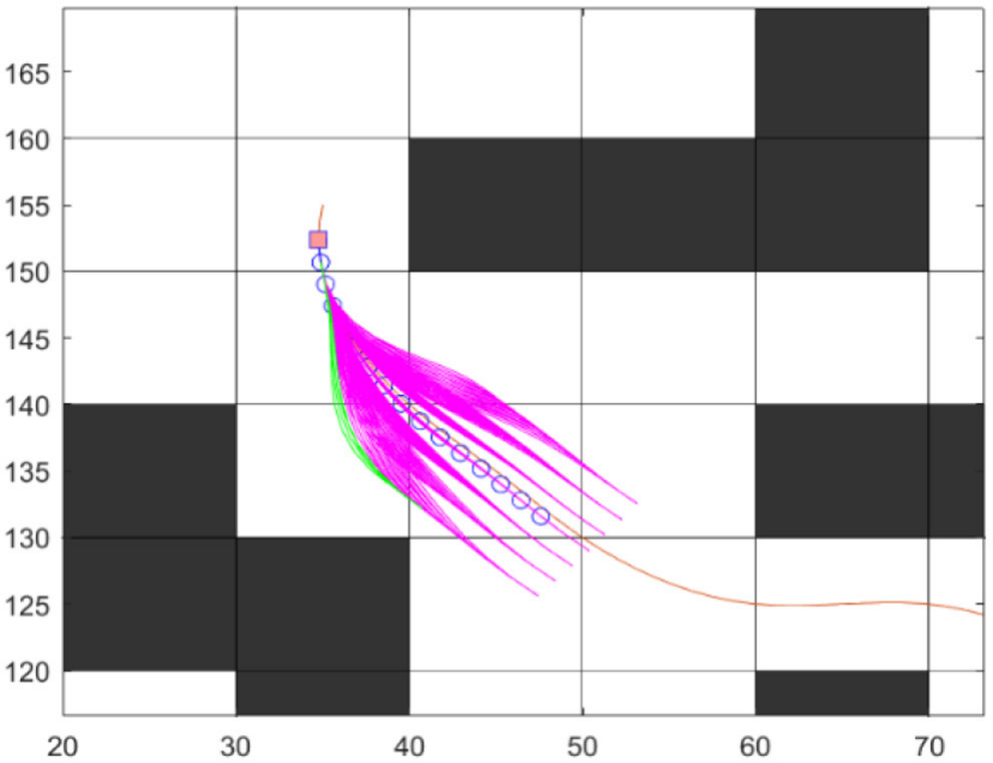
Local Trajectories from lateral and longitudinal movements.

The obtained global route consists of smoothly distributed waypoints produced through arc-length parameterization. To maintain its position on reference route and avoid obstacles, robot has to move along offset of center line and offset distance represented with *d*. Arc-length *s* is taken as parameter to localize the position of robot at each waypoint on global route. Both *s* and *d* has been considered as lateral and longitudinal coordinates. For each forward move on reference route, a set of lateral and longitudinal coordinates are generated separately and unified to form a set of trajectories as shown in Figure 3. Time parameterization is used in producing lateral and longitudinal coordinates. In Werling et al. (2010), Quantic and quartic polynomial coefficient are derived to calculate a set of longitudinal and lateral trajectories with different final times and different final states (relative to the reference line). At initial ε_0_ and final ε_*t*_ states lateral and longitudinal movements are considered as:

(9) $$\begin{array}{r} \varepsilon_{0}=\left[\varepsilon_{0},{\overset{∙}{\varepsilon }}_{0},{\ddot{\varepsilon }}_{0}\right],\;\varepsilon_{t}=\left[\varepsilon_{t},{\overset{∙}{\varepsilon }}_{t},{\ddot{\varepsilon }}_{t}\right] \end{array}$$

Furthermore, it can be minimized through considering square of jerk *J*_*t*_ with defined time integral from *t*_0_ to *t*_1_.

(10) $$\begin{array}{r} J_{t}\left(\varepsilon \left(t\right)\right):=\int_{t_{0}}^{t_{1}}{\overset{\mathrm{...}}{\varepsilon }}^{2}\left(\tau \right)d\tau \end{array}$$

cost of trajectory *C* in constraint free environment is based upon sum of individual cost of lateral (*C*_*d*_) and longitudinal (*C*_*s*_) components, respectively.

(11) $$\begin{array}{r} C=k_{j}J_{t}+k_{t}g\left(T\right)+k_{p}h\left(\varepsilon_{t}\right) \end{array}$$

*T* is referred as time interval of transforming state ε_0_ to ε_*t*_ and g and *h* has been assumed arbitrary functions with parameters *k*_*j*_, *k*_*t*_ and *k*_*p*_. At first, lateral coordinates have been generated to specify the vehicle steering direction and to maintain offset distance parallel to center line. Initial lateral position of robot is *d*_0_, ${\overset{\cdot }{d}}_{0}$, and ${\overset{.}{d}}_{0}$ refers to initial lateral velocity and acceleration, respectively. In set of final trajectories, lateral movements have been unified with longitudinal movements. In this content, Quantic polynomial (Rathgeber et al., 2015) has been used to get smooth lateral deviations suitable to longitudinal terminal manifolds in different modes of operations. ${\overset{\cdot }{d}}_{0}$ and ${\ddot{d}}_{0}$ has taken 0 in starting, as the robot has to move parallel with center line.

(12) $$\begin{array}{r} D_{0}=\left[d_{0},{\overset{.}{d}}_{0},{\overset{\mathrm{..}}{d}}_{0},T\right]=\left[d_{0},0,0,T\right] \end{array}$$

Longitudinal coordinates have been utilized to localize the robot on reference line. At each forward move on global route, offset distance from centered waypoint to robot position is considered as *d*. Waypoint robot covers arc length *s* and estimate *s*_*t*_ position in time interval T paired with set of lateral movement using quartic polynomials (Guan et al., 2005). Initial longitudinal state of robot is $S_{0}=\left[S_{0},{\overset{\cdot }{S}}_{0},{\ddot{S}}_{0}\right]$ and time *t*. After T interval it estimates terminal state $S_{t}=\left[{\overset{\cdot }{S}}_{t},{\ddot{S}}_{t}\right]$ and time changes to *t*_*t*_. Longitudinal trajectory generation can be achieved efficiently with quartic polynomials through varying the Δṡ_0_ and *T*.

(13) $$\begin{array}{r} {\left[{\overset{\cdot }{s}}_{t},{\ddot{s}}_{t},T\right]}_{0t}=\left[\left[{\overset{\cdot }{s}}_{d}+\Delta {\overset{\cdot }{s}}_{0}\right],0,T\right] \end{array}$$

In case of moving robot with constant velocity ṡ_*d*_ it requires to a constant velocity instead of defining position to define its motion along reference path. The functional cost can be minimized through quartic coefficient.

(14) $$\begin{array}{l} C_{d}=k_{j}J_{t}\left(d\left(t\right)\right)+k_{t}\left(T\right)+k_{d}h\left(d_{t}\right) \end{array}$$

(15) $$\begin{array}{r} C_{s}=k_{j}J_{t}\left(s\left(t\right)\right)+k_{t}\left(T\right)+{k_{s}\left[{\overset{\cdot }{s}}_{t}+{\overset{\cdot }{s}}_{d}\right]}^{2} \end{array}$$

In order to achieve a unified pair of trajectories on reference path, each lateral and longitudinal set has been passed through initial check. And trajectories coupled with unsuitable lateral and longitudinal accelerations has been removed based on the values of $\ddot{d}$ and $\ddot{s}$. The final cost of each optimal trajectory has been minimized through summing individual costs *C*_*d*_ and *C*_*s*_ (Werling et al., 2010). Similarly, every trajectory has been verified with respect to its cost value and minimum jerk *J*_*t*_.

The final cost of each optimal trajectory has been minimized through summing individual costs *C*_*d*_ and *C*_*s*_. In constraints avoidance scenario, a safety distance has defined to keep away collision. Every trajectory has been checked with respect to its cost value. With this regard, the heading θ(*t*), curvature κ(*t*), velocity *v*(*t*), and acceleration *a*(*t*) has been taken in consideration to give underlying control of actuators. Therefore, curvilinear coordinates have been transformed to Cartesian coordinates as in Werling et al. (2012).

## Simulation Results

**Figure 4:**
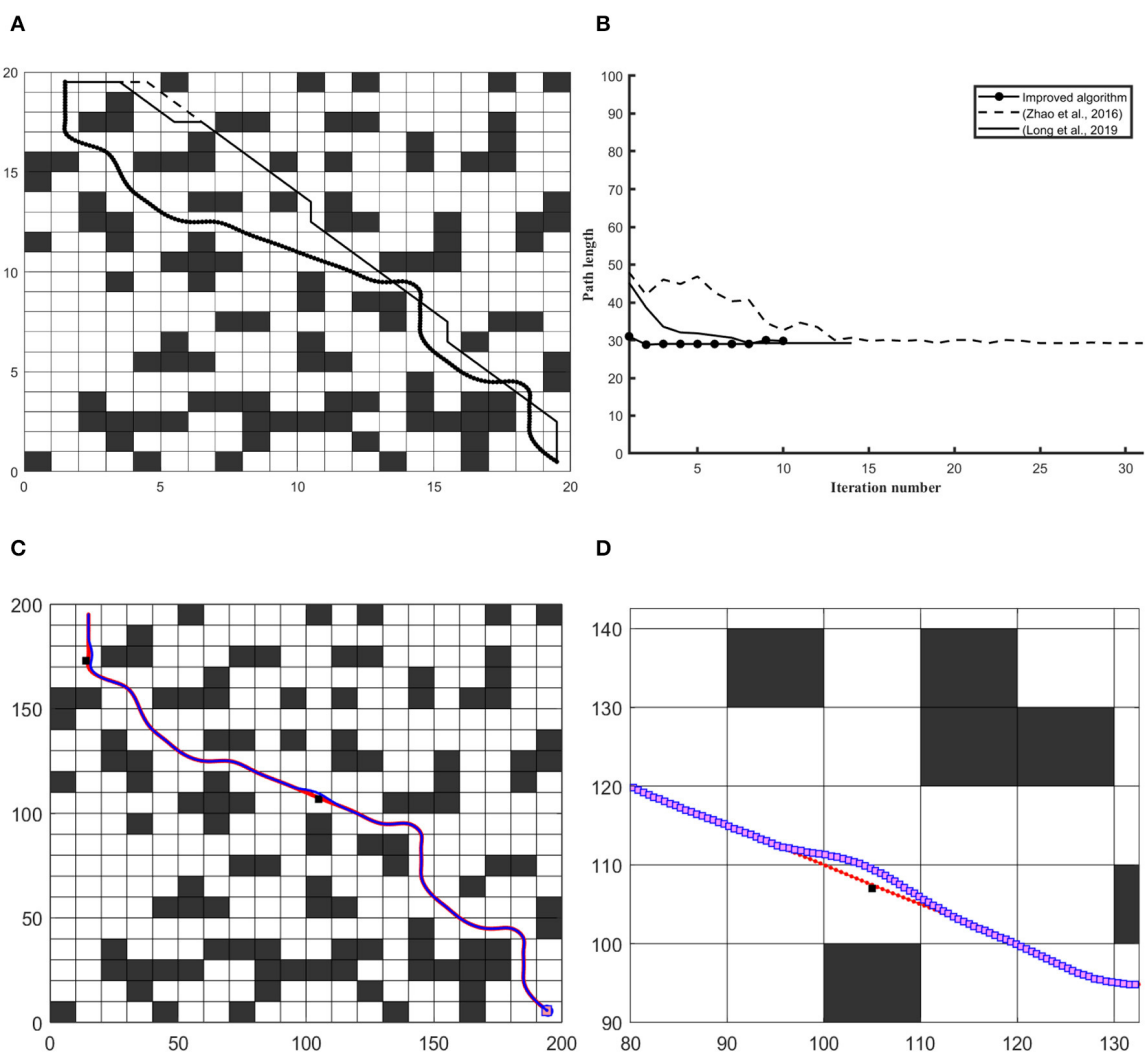
The simulation results on 20 × 20 and 200 × 200 workspace in a common map. **(A)** Differentiates the quality of the global path **(B)** Convergence graph of iteration vs. path length. **(C)** Differentiates robot trajectory with respect to a reference frame of the curvilinear path under the unknown obstacle. **(D)** Shows a close look at obstacle avoidance and robot trajectory vs. obstacle.

Path length has been considered a key element in the optimization of the global path planning problem. With the shortest and path smoothness, the robot consumes less amount of fuel to perform the task in minimum time. This paper improves both features to achieve an optimal trajectory. To verify the efficiency of the combined approach, simulations have been performed in MATLAB on different complex maps and robot size has assumed smaller than grid resolution. In first section, optimal global path has been shown and compared with previous versions of ACO in sense of path consistency, smoothness and safety. In second part, the results presented under both pre-defined and unknown static constraint environments along with details have been given in Table 6. In Figure 4, the algorithm has been applied on common map with static constraint environment in 20 × 20 workspace and compared with Long et al. (2019) and Zhao et al. (2016). The figure shows that the obtained path has consistency in order to localize the robot along global route, as well as, the graph in Figure 4B verifies the efficiency of algorithm in reducing No. of iterations along with shortest distance considering safety and comfort. In Figure 4C, reference global path is represented through red dotted line and blue dotted line representing robot's trajectory under placement of unexpected static obstacles.

**Table 6:** Simulation results.

	**Common map**			**Tunnel map**			**Through map**			**Baffle map**		
Algorithm	1	2	3	1	2	3	1	2	3	1	2	3
Number of sharp bends	10	10	0	12	10	0	–	13	0	–	10	0
Average path length	29.45	29.38	29.23	38.48	38.12	37.99	–	51.84	52		41.48	41.89
Number of iterations	33	12	9	35	16	10	–	40	11	–	16	10
Time of reference trajectory generation (sec)	7.26	4.89	1.49	20.62	17.97	1.601	–	88.20	1.778		10.98	1.39
Number of unknown obstacles	–	–	2	–	–	2	–	–	5	–	–	5
Time taken by robot to respond unknown constraints (millisec)	–	–	60	–	–	60	–	–	60	–	–	60

^*^ Algorithm.

*1-described in Zhao et al. (2016)*.

*2-described in Long et al. (2019)*.

*3-Derived approach in this paper*.

**Figure 5:**
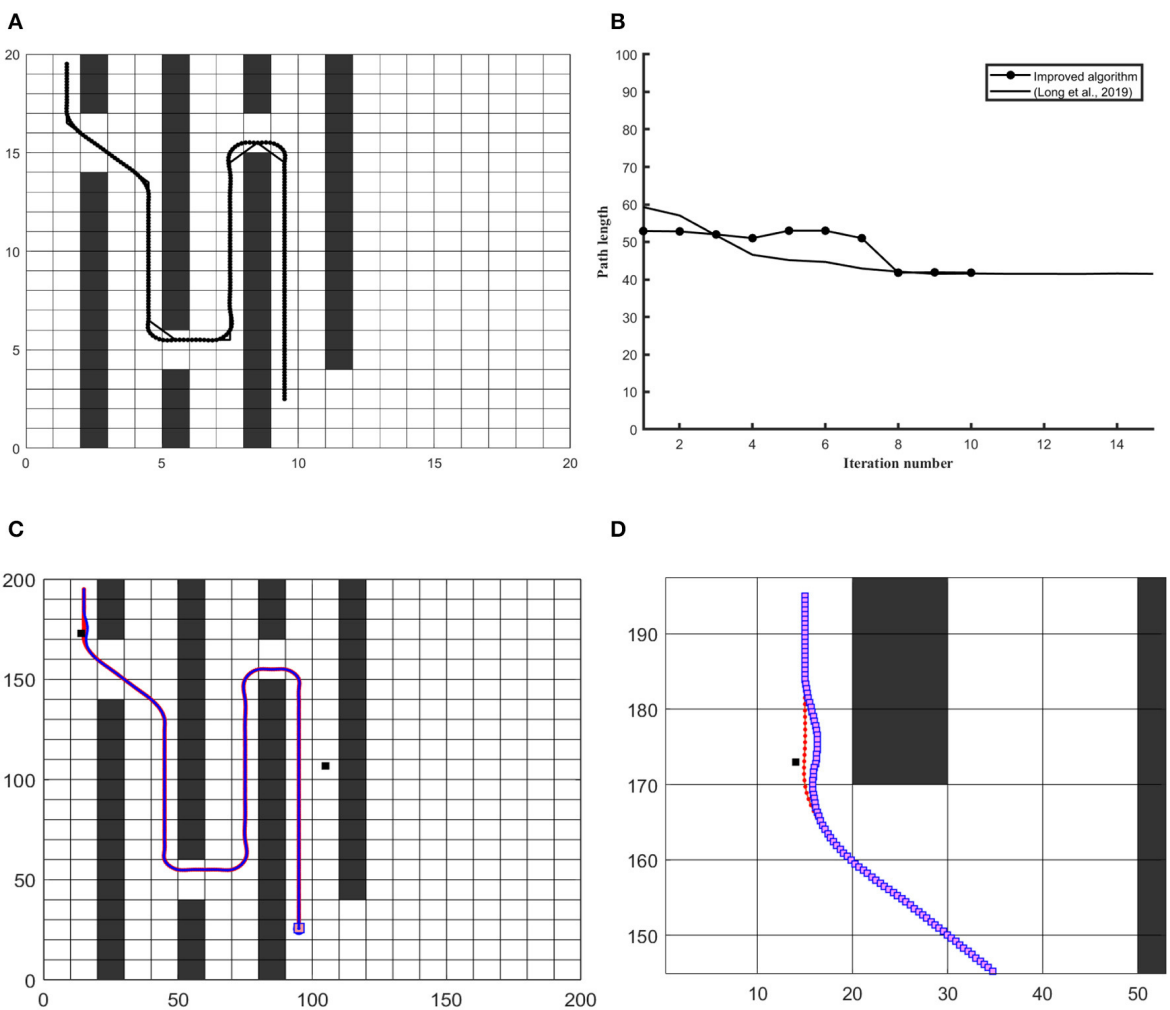
The simulation results on 20 × 20 and 200 × 200 workspace in the baffle map. **(A)** Differentiates the quality of the global path **(B)** Convergence graph of iteration vs. path length. **(C)** Differentiates robot trajectory with respect to a reference frame of the curvilinear path under the unknown obstacle. **(D)** Shows a close look at obstacle avoidance and robot trajectory vs. obstacle.

In Figure 5, the baffle map has been selected to optimize the global path with a static constraints environment in the workspace. This approach gives an optimal path in baffle map with smoothness and enhanced path visibility, that assists the robot to localize and navigate efficiently, rather than (Long et al., 2019). In Figure 5B Vertical axis represents path length and the horizontal axis represents the number of iterations, respectively. Figure 5A differentiate the quality of path and Figure 5B shows that the number of iterations is lower than (Long et al., 2019) and, the computation time of this approach has also reduced compared with previous versions.

**Figure 6:**
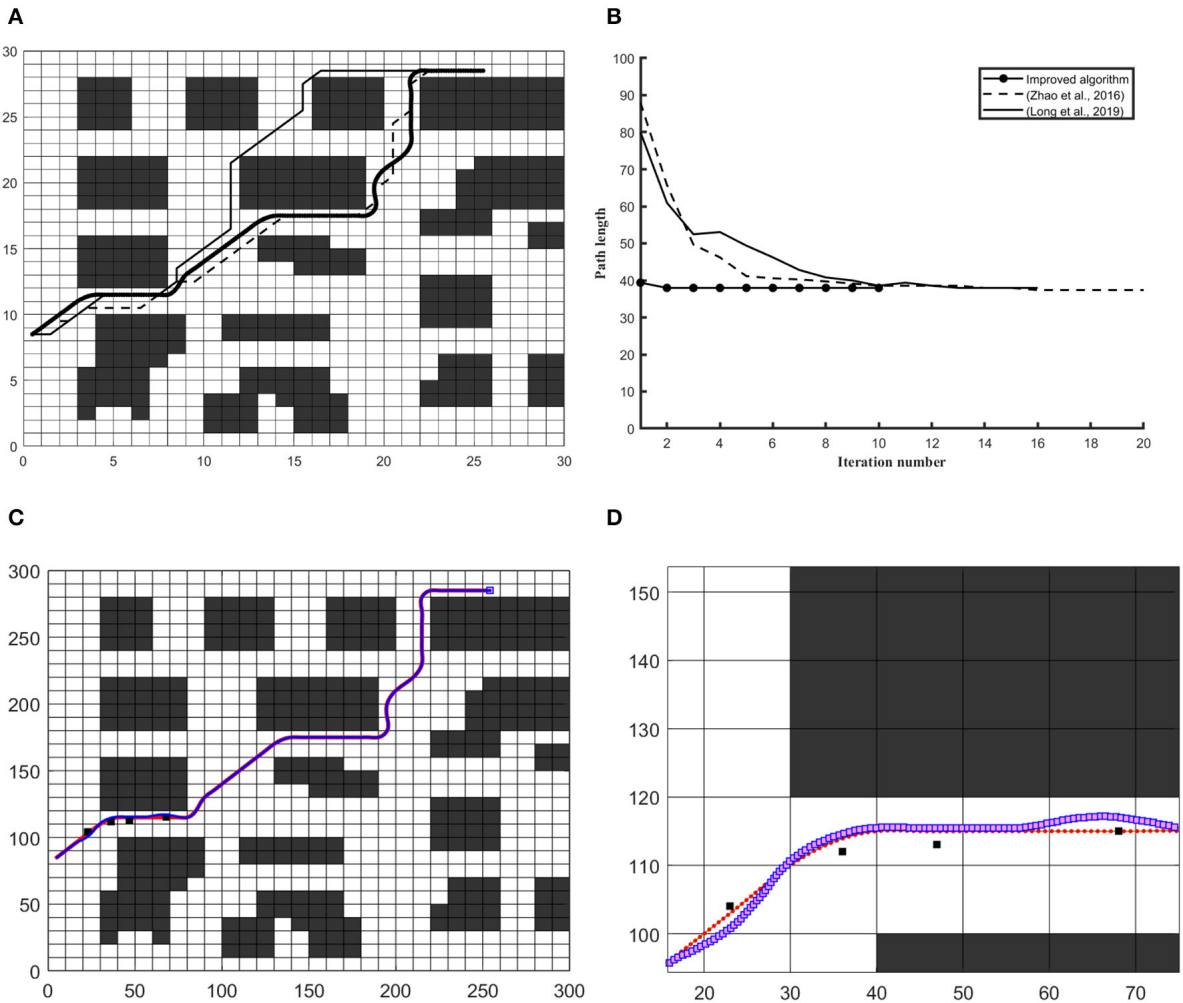
The simulation results on 30 × 30 and 300 × 300 workspace in the tunnel map. **(A)** Differentiates the quality of the global path **(B)** Convergence graph of iteration vs. path length. **(C)** Differentiates robot trajectory with respect to a reference frame of the curvilinear path under the unknown obstacle. **(D)** Shows a close look at obstacle avoidance and robot trajectory vs. obstacle.

In Figure 6, algorithm has been applied on tunnel map. In Figure 6A Global route has shown to differentiate the results and Figure 6B further confirms efficiency of this approach with respect to finding optimal global path in 30 × 30 tunnel map. In order to deal with static and dynamic path planning, a 300 × 300 workspace is created. In Figure 6C, reference path is shown with red dotted line and robot trajectory along reference path has been presented with blue dotted line and Figure 6D gives a close view of obstacle avoiding trajectory.

**Figure 7:**
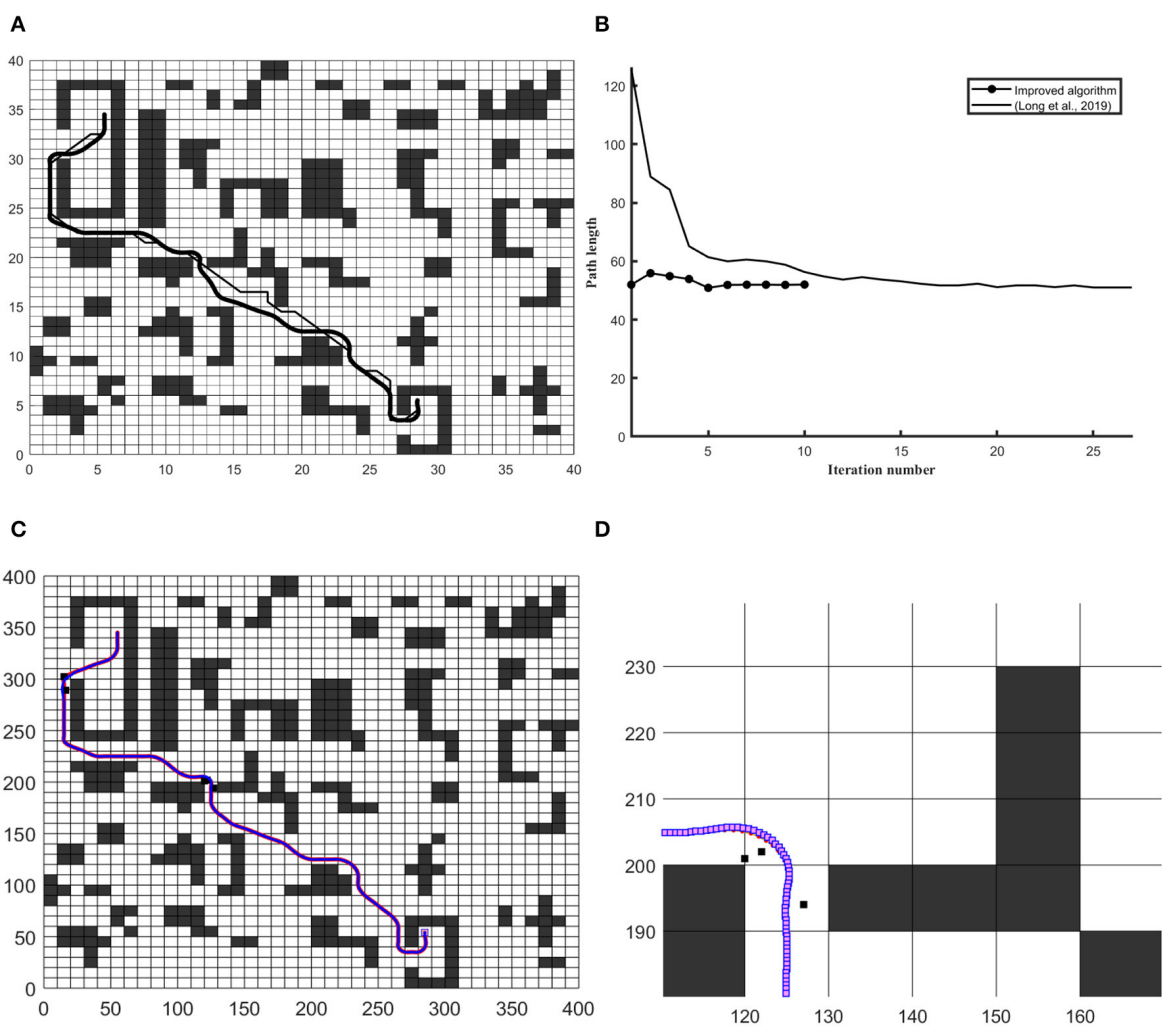
The simulation results on 40 × 40 and 400 × 400 workspace in through map. **(A)** Differentiates the quality of the global path **(B)** Convergence graph of iteration vs. path length. **(C)** Differentiates robot trajectory with respect to a reference frame of the curvilinear path under the unknown obstacle. **(D)** Shows a close look at obstacle avoidance and robot trajectory vs. obstacle.

Similarly, Figure 7 demonstrate the efficiency of this derived approach in 40 × 40 search space to solve static and dynamic path planning problems in grid-based environment. The obtained path in Figure 7A has been compared with Long et al. (2019) and the quality of derived path founds suitable to robot motion. The workspace has been increased to 400 × 400 in order to optimal trajectory in known and unexpected static constraints environment. In Figure 7C, robot's trajectory has been represented with blue dotted line and reference path represented with red dotted line. It indicates that robot follows reference route at unconstraint situation, and at some places robot's trajectory differs from reference route to avoid obstacle appearing ahead of it.

## Conclusion and Future Work

This paper introduces a combined approach for a mobile robot to deal with path planning problems in static and dynamic constraints environment and will be applied on mobile robot in real world environment. In the first part, global reference route is obtained through A* Multi-directional algorithm and improved ACO. MDP model based on novel reward policy has been introduced to evaluate the global path points generated in grid-based environment. Arc-length parametrization generates a curvilinear global route among obtained waypoints. In order to deal the environment with dynamic constraints, a set of lateral and longitudinal coordinates have been generated to maintain a suitable offset distance from global reference path and to produce a set of trajectories along global route. In addition, a cost policy is defined to choose the constraints-free smooth trajectory. In this way, an optimal approach is derived to deal with the complexity of different maps considering safety, path consistency and smoothness of trajectories compared to other grid-based approaches in complex-constraints environment. The simulations have been performed in MATLAB to verify its efficiency.

Although, the ability and functionalities of this approach in GPP and LPP can be extended further to cover verity of mobile robot applications and to avoid dynamic moving obstacles.

## Data Availability Statement

All datasets generated for this study are included in the article/supplementary material.

## Author Contributions

HA proposed this contribution. DG and MW verified and conclude simulation results. XD gave suggestions in manuscript writing. All authors contributed to the article and approved the submitted version.

## Conflict of Interest

The authors declare that the research was conducted in the absence of any commercial or financial relationships that could be construed as a potential conflict of interest.
